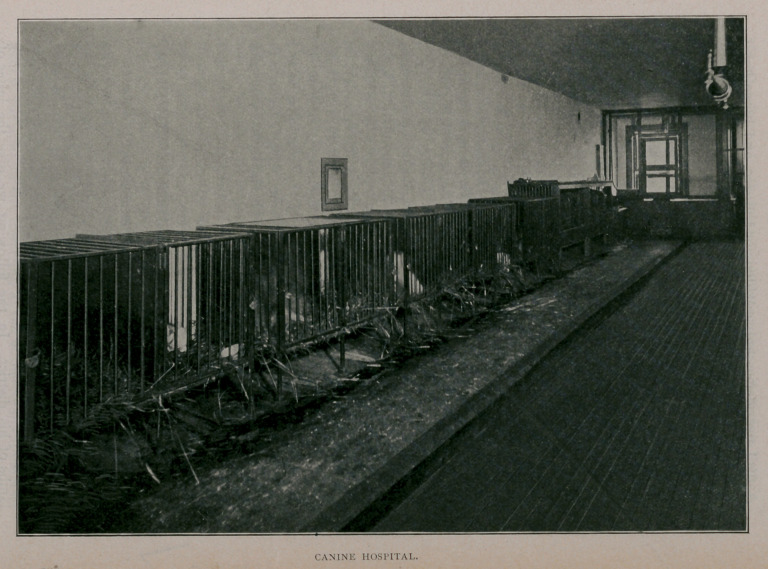# New York College of Veterinary Surgeons

**Published:** 1895-07

**Authors:** 


THE JOURNAL
OF
COMPARATIVE MEDICINE AND
VETERINARY ARCHIVES.

VOL. XVI. *JULY*, 1895. No. 7.

### NEW YORK COLLEGE OF VETERINARY SURGEONS.

UNDER the title of Chapter 269, "An Act for the incorporation of the New York College of Veterinary Surgeons" was passed and signed at Albany on April 6, 1857, establishing a board of nine incorporators, who were intrusted with the organization of a veterinary school, with the right to hold property not to exceed one hundred thousand dollars, and which was to be conducted solely in trust, for the gain of no individual, and all funds or property to be used for promoting medical science and instruction.

No active steps were taken in the development of the college for several years, when, in 1862, further legislation was obtained. Chapter 346, an Act to amend Chapter 269, was passed, adding fourteen trustees, making twenty-three in all.

In September, 1862, a meeting of the trustees was called, at which E. G. Rawson, Esq., was appointed temporary chairman, and Charles H. Birney, Esq., was elected the first president. Dr. John Busteed was elected professor of anatomy and surgery, president of the faculty, and corresponding secretary of the corporation. A month later one of the trustees resigned, and Dr. Busteed was elected a trustee in his place. Regular meetings were held twice a month, for the purpose of raising funds. But that period, of war time, was not a favorable one for obtaining generous gifts for any ordinary purpose.

In May, 1863, Drs. Rawson, Busteed, and Alfred Roe, Esq., of the trustees, were appointed a committee to attend the veterinary meeting in Philadelphia, which was the origin of the organization of the United States Veterinary Medical Association.

During the next year subscriptions gradually accumulated, and on October 31, 1864, the infirmary of Dr. Liautard, 179 Lexington Avenue, was selected as the site for the college, and was purchased from him.

Two interesting paragraphs occurred in the papers and arrangements for taking possession of the property, showing the local condition of money and commerce at the time The first, a reference in the lease to gold and the great fluctuating value of all money; the second, that the trustees should purchase the food for the winter at once in large quantities, as an economy, on account of the approaching cold and close of navigation, all freight being then brought to New York by canal-boats.

On November 23d, 1864, the college was declared ready for the reception of patients and for clinical instruction. Dr. Liautard was appointed superintendent of the hospital. In March, 1865, Dr. Busteed presented to the trustees a copy for the first announcement for the regular course of lectures, which was approved. On October 4, 1865, Augustus Whitlock, Esq., was elected president of the trustees.

The college opened on November 6th with the following faculty; the members, however, appear to have assumed their duties without regular appointment: Anatomy and operative surgery, A. F. Liautard, M.D., V.S.; physiology and surgical pathology, A. Large, M.D., M.R.C.V.S.; pathology of the horse and other domestic animals, A. S. Copeman, V.S.; materia medica and therapeutics, J. Busteed, M.D.; organic chemistry and practical use of the microscope, A. S. Copeman, V.S.; clinical instruction, Drs. Liautard, Large, and Copeman.

In January, 1866, the use of the lecture-room of the college was granted to the New York Veterinary Society for its meetings.

In May, President Whitlock died, and Charles H. Birney was elected to fill the chair for the remainder of the year, until the regular election, when in September Eben Mason was elected president. In March, 1867, Dr. Busteed handed in his resignation as professor of anatomy and surgery, which was accepted, and he was appointed emeritus professor of the same chair. Dr. Liautard, who had been giving the lectures upon anatomy and surgery, was appointed professor of that chair, and Dr.

Large was appointed to the chair of physiology and pathology, which had been in his charge since the opening of instruction. It was resolved that the first diploma of V.S. should be conferred upon Dr. Busteed.

The following November, Henry Bergh, the president of the Society for the Prevention of Cruelty to Animals, was elected a trustee. At about this period there was some attempt made to subvert the purpose of the school from veterinary instruction to that of a school of human medicine, which, however, did not succeed.

An active attempt was made by the trustees of the college at this time to obtain recognition by the United States Government and to ameliorate the condition and status of the veterinary surgeons in the army. A petition was prepared and sent to General Grant, asking his approval, in order that it should be submitted to Congress. But it received no further attention than an unfavorable reply.

In February, 1868, the trustees of the college prepared and gave a reception to Dr. Gamgee, president of the Albert Veternary College, London. He had been sent to the United States to investigate cattle diseases.

In September, 1868, William Palen, Esq., was appointed president. In October, 1870, Alexander J. Cotheal was elected president, and Dr. James L. Robertson, one of the first graduates of the New York College of Veterinary Surgeons, and Dr. F. L. Satterlee were appointed lecturers in the college.

During the next few years the New York College of Veterinary Surgeons ran a quiet existence, gradually increasing in the number of its students and in the size of the clinic and its hospital, but did not make any marked headway.

Internal disturbances arose between Dr. John Busteed, representing both the trustees and as president of the faculty, and other members of the trustees and faculty. There seems not to have been a perfect accord with either body, and some bitter charges and counter-charges appear to have taken place between the trustees and the faculty. On February 10th, 1875, Dr. John Busteed was elected president of the board of trustees. The trouble of the last few years assumed more definite shape, and Court injunctions were obtained, interfering with the action of the board.

On March 10, 1875, an almost wholesale resignation of the faculty was sent in, signed by Drs Liautard, Weisse, Stein,

- HOSPITAL. -

Robertson, and Satterlee. The dissensions seem to have grown more bitter during this year, and no lectures were given during the following session.

On October 6, 1875, Dr. A. J. Cotheal was elected president. In the spring following, April 10, 1876, Dr. John Busteed died, and at the December meeting it was decided to sell the college buildings.

On June 7, 1877, E. G. Rawson, A.M., M.D., was elected president of the college faculty, which was then reorganized as follows: Professor of general and comparative anatomy, D. C. Comstock, M.D.; professor of physiology and chemistry, Thomas M. Hawkins, M.D.; professor of materia medica and therapeutics, E. S. Bates, M.D.; professor of theory and practice, R. S. Finley, M.D.; professor of surgery and surgical pathology, J. A. Going, M.R.C.V.S.; professor of obstetrics, Thomas H. Skinner, M.D.

On September 18, 1878, Dr. William T. White was elected a member of the Board of Trustees; at the following meeting he was elected member of the medical committee, and on February 6, 1879, John M. Guiteau, Esq., was elected a trustee, both of whom proved to be valuable members of the board, both in the direct interest which they took in the college and in their constant care in the protection of its privileges.

On September 19, 1883, Dr. W. T. White, was elected president of the board of trustees, to the interest of which office he was devoted until the time of his death, in 1894.

In 1880 Dr. White purchased a two-story building at 332 East Twenty-seventh Street. He added another story and made extensive alterations to adapt it for the uses of the college. This building he fitted with furniture and the necessary apparatus, and had it prepared for use by October, 1880. He rented this building to the New York College of Veterinary Surgeons.

The college then moved into its new building, 332 East Twenty-seventh Street, and became much more active in its work. It was carried on in this location for the next half-dozen years, making steady growth in the proportion of its museums and the numbers of its students.

On September 17, 1894, Dr. William T. White died, just as he had reached the point which looked to the great improvement of the college. At the November meeting of the same year Dr. Herman M. Biggs was elected a trustee, and at the February meeting, 1895, he was elected president of the board.

As the new president recognized the entire inadequacy of the present building, steps were taken immediately looking toward an improvement. The property in the rear was purchased and a beginning was made in the necessary changes; but it was soon found that the very considerable expenditures necessary would give at best only an unsatisfactory building in a yet more unsatisfactory site. The neighborhood was poor in itself, as it had become too far removed from the centre of the city, which had gradually grown northward and was inaccessible to that class of practice which the hospital and school should expect.

After a considerable search a building at 154 East Fifty-seventh Street was found, and it was apparent, immediately, that no amount of search would be likely to result in the choice of a site more admirably adapted for the necessities of school and hospital. A central location, a wide and frequented street, a good public neighborhood, were all won by the school by the move.

The building is a five-story brick with an "L" at the rear. The first effort was to secure the most thorough sanitation of the building; next to study the convenience of every part. The two lower floors are asphalted and devoted to the hospital for horses; new box and open stalls were constructed upon the most approved plans, and these are equipped with the latest approved stable furniture. Above the hospital are placed the offices, pharmacy, chemical laboratory, lecture and reading-rooms; up another flight are placed the museum and histological and bacteriological laboratories, and an abundant space is devoted to the dissecting-room; finally, the top floor is given up to a dog hospital, which is especially fortunate in having the use of the "L" for an outdoor run, giving an area of eighteen hundred feet. An "L" shaft running through the middle of the building from top to bottom, furnishes a ready means of carrying up the material for dissection, and other freight, and serves as well for additional means of hospital ventilation and light.

Especial mention should be made of the operating-room, constructed with special reference to the practice of aseptic surgery, well-lighted, and provided with sterilizers, an operating-table, hot and cold water, and all conveniences.

A forge is supplied with all necessary tools, etc., for the manipulation of the foot and manufacture of shoes. For immediate convenience a boiler was added, of sufficient capacity to supply hot water in every part of the building.

CHEMICAL LABORATORY.

Small pharmacies are provided for the dog and horse hospitals, sufficient for all needs, while the large pharmacy, devoted to the purposes of instruction, is supplied with all drugs and prescriptions ordinarily used in veterinary medicine.

The lecture-room is fitted with two hundred seats. It is well lighted and ventilated from above as well as with windows at side and end.

An important subject which engaged the attention of the trustees and faculty, who have throughout worked in unison, is the improvement of the character and amount of instruction given. It was clear, of course, that the first step was to grade the course, in compliance with the demands of the United States Veterinary Medical Association. The course was extended to a three-years' graded one, and this change was announced in the first circular published after the reorganization.

Next in importance in the new order was the enlargement of the faculty by the addition of well-known specialists in the various departments of veterinary medicine. Time was needed to gain certainty of judgment in the rapid lines of change, but since the reorganization the faculty has been increased and strengthened by the addition of several valuable members; Dr. R. W. Hickman, graduate and formerly demonstrator of anatomy in the University of Pennsylvania, who is now in charge of the New York department of the Bureau of Animal Industry; Dr. W. Lillman, late graduate of Giessen and Berlin; Rush Shippen Huidekoper, M. D., Veterinarian (Alfort), who organized the Veterinary Department of the University of Pennsylvania and was the first dean of that college. Dr. Huidekoper also lectured two years at the American Veterinary College and is the President of the New York State Veterinary Medical Society. He is the author of several works on veterinary medicine.

Among the graduates of the New York College of Veterinary Surgeons appear the names of a number of men who have become celebrated in the veterinary profession throughout the whole of the United States. Among the first was James L. Robertson, who for the next few years was an instructor in the college, and in more recent years has been the popular teacher at the American Veterinary College, and who is also well known as the treasurer of the United States Veterinary Medical Association.

Among other well-known graduates have been, Harry D. Gill, present dean of the college; James Hamill, the specialist on diseases of the foot; Patrick Burns, of New York; Roderick

CANINE HOSPITAL.

A. McLean, of Brooklyn; William H. Birch, of Philadelphia; James H. Ferster, of New York, now lecturer at the college; Robert W. Finley, of New York City; Charles B. Michener, for years the well-known secretary of the United States Veterinary Medical Association and professor for some years at the American Veterinary College and at the New York College of Veterinary Surgeons. He recently died, after having held an important position under the Government in the Bureau of Animal Industry, at Washington, D. C. Other graduates of the college, too numerous to mention, have made names for themselves and reputation for the college in public life.

Among the well-known names which have appeared in the faculty are those of: A. F. Liautard, M.D., V.S.; A. Large, M.D., M.R.C.V.S.; A. S. Copeman, V.S.; J. Busteed, M.D.; J. L. Robertson, M.D.; F. L. Satterlee, M.D.; Samuel R. Percy, M.D.; Alex. W. Stein, M.D.; Edmund G. Dawson, A.M., M.D.; D. C. Comstock, M.D.; Thomas M. Hawkins, M.D.; E. S. Bates, M.D., R. S. Finley, M.D.; J. A. Going, M.R.C.V.S.; Thomas H. Skinner, M.D.; F. H. Stephens, M.D.; J. B. Coleman, M.R.C.V.S.; J. M. Heard, M.R.C.V.S.; L. McLean, M.R.C.V.S.; L. V. Plageman, M.R.C.V.S.; A. S. Heath, M.D.; W. S. Gottheil, M.D.; Alex. Lockhart, M.R.C.V.S.; R. A. McLean, H. D. Gill, V.S.; T. Robertson, M.R.C.V.S.; J. A. Breakell, M.D., V.S.; H. M. Biggs, M.D.; Charles B. Michener, V.S.; E. F. Brush, M.D.; W. R. Ballou, M.D.; J. S. Hopkins, M.D.; Charles L. Allen, M.D.; J. H. Huddleston, M.D.; George P. Biggs, M.D.; O. E. Busener, V. S.; A. C. Hasslock, V.S., and Harvey T. Potter, V. S.

## Figures and Tables

**Figure f1:**
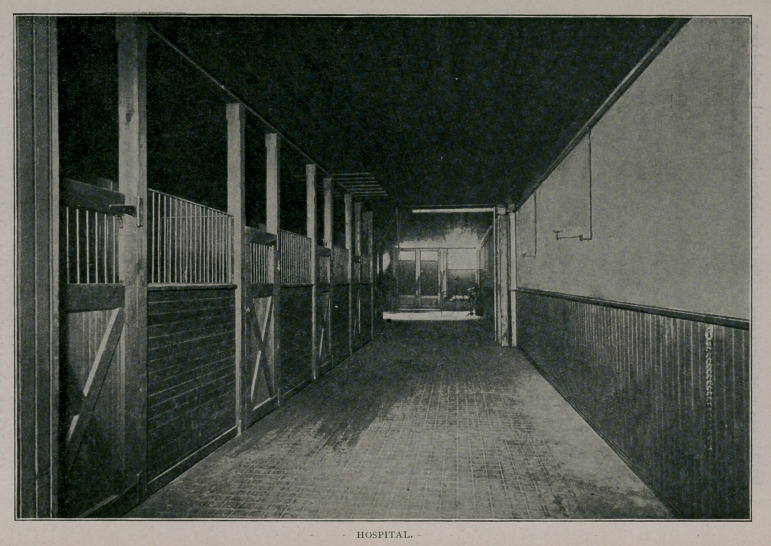


**Figure f2:**
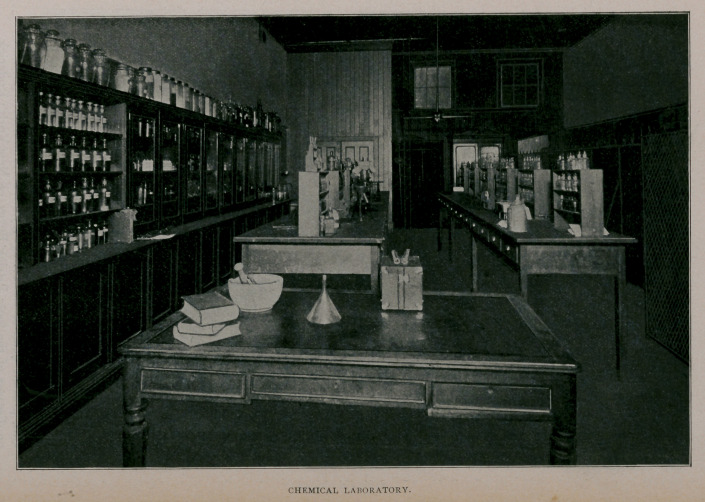


**Figure f3:**